# Apparent Diffusion Coefficient of Diffusion-Weighted Imaging in Evaluation of Cervical Intervertebral Disc Degeneration: An Observational Study with 3.0 T Magnetic Resonance Imaging

**DOI:** 10.1155/2018/6843053

**Published:** 2018-02-18

**Authors:** Pinjie Chen, Chunlei Wu, Minghua Huang, Guangjian Jin, Qinglei Shi, Zhihua Han, Chun Chen

**Affiliations:** ^1^Department of Traditional Chinese Medicine (TCM) Orthopedics, The First Affiliated Hospital, Wenzhou Medical University, Wenzhou, China; ^2^Department of Radiology, Navy General Hospital, Beijing, China; ^3^Department of Orthopedics, The First Affiliated Hospital, Wenzhou Medical University, Wenzhou, China; ^4^Siemens Ltd., China Healthcare Sector MR Business Group, Beijing, China; ^5^Experimental Trauma & Orthopedic Surgery, Goethe University Frankfurt, Frankfurt am Main, Germany; ^6^Department of Orthopedics, The First Affiliated Hospital, Anhui Medical University, Hefei, China; ^7^Engineering Research Center of Clinical Functional Materials and Diagnosis & Treatment Devices of Zhejiang Province, Wenzhou Institute of Biomaterials and Engineering, Wenzhou, China

## Abstract

**Aims:**

To investigate the correlation between the apparent diffusion coefficient (ADC) value and cervical intervertebral disc degeneration in adult symptomatic patients.

**Methods:**

A total of 52 symptomatic and 40 healthy volunteers were included. DWI and routine MRI examinations were performed to their cervical spines. The cervical discs (from C2-C3 to C6-C7) were graded according to the Pfirrmann grading system, and ADC values of the nucleus pulposus (NP) were measured. Differences of the ADC values between different genders and anatomic levels were analyzed; the correlation between the ADC value and the Pfirrmann grade was investigated. The cut-off ADC values of each Pfirrmann grade were calculated.

**Results:**

The mean ADC value of the NP decreased with increasing Pfirrmann grade (I–V) upon both patients and asymptotic volunteers. The ADC value decreased descendingly from C2-C3 to C5-C6 (*P* < 0.05) and then increased at C6-C7 (*P* < 0.05). Additionally, the comparison of the ADC values between different genders achieved statistical significance at each anatomical level (*P* < 0.05), except at C6-C7 (*P* > 0.05). Significant negative correlations between the ADC value and either age or Pfirrmann grade were observed.

**Conclusions:**

Our preliminary findings suggest that the ADC value obtained by DWI can provide a reliable indicator to evaluate the cervical disc degeneration.

## 1. Introduction

Cervical intervertebral disc degeneration (IVDD) is the main cause of spine disorders resulting in neck pain and upper limb numbness, with the age increases the incidence of cervical degenerative diseases also rises [1]. Cervical IVDD is described as an abnormal, cell-mediated response to progressive structural failure of the discs [2]. Late stage or severe IVDD will progress to herniated disc and cervical spinal stenosis, requiring invasive treatments with heavy financial burden. Thus, early diagnosis and intervention of IVDD are important to eliminate symptoms and to treat the disc degeneration.

Magnetic resonance imaging (MRI) is a useful modality in the assessment of IVDD, since it is highly sensitive to the water content within the tissue [3]. T2-weighted (T2-WI) and T1-weighted (T1-WI) MRI are the most common techniques to examine the health of intervertebral discs. Based T2-WI imaging, Pfirrmann grading system had been developed to evaluate the severity of disc degeneration, according to the signal intensity and morphological shape on the T2-WI images. Such grading system has been widely accepted and used for decades. However, drawbacks exist inherently in traditional MRI techniques at detecting biochemical changes within the disc [4], therefore restricting their applications for early diagnosis of IVDD.

To understand the biochemical changes in different types of tissue, many quantitative functional MRI techniques have been developed, such as T2 mapping, T2 star, and diffusion-weighted imaging (DWI). Those techniques were previously utilized to investigate biochemical changes in human and animal IVDs [4–6]. Among those techniques, DWI is demonstrated to be able to reveal pathophysiological status within intervertebral discs [4]. In particular, DWI can provide valuable information of the biochemical content in the disc by detecting random motion of water molecules in intracellular or extracellular fluid [6, 7]. Based on DWI data, apparent diffusion coefficient (ADC) can be calculated to qualify the degree of water diffusion within the disc.

However, application of DWI in diagnosis of IVDD is hindered by an important technical restriction. Specifically, single-shot echo-planar imaging (SS-EPI) is the routine sequence for clinical DWI tests [8], but there are many disadvantages of the SS-EPI, including susceptibility artifacts that manifest as image blurring, signal dropout, or geometric distortion [9]. Most currently, readout-segmented echo-planar imaging (RS-EPI) has been suggested as a better alternative to SS-EPI because of its reduced distortion [10]. Furthermore, the RS-EPI was proved to be better regarding the qualitative image quality, like signal-to-noise ratio (SNR), lesion contrast, and contrast-to-noise ratio (CNR) [11]. The two-dimensional navigator-corrected RS-EPI sequence, RESOLVE (REadout Segmentation Of Long Variable Echo trains), is the latest RS-EPI technique. It is able to reduce distortion caused by susceptibility, respiration, motion, and pulsations and increase spatial resolution for higher quality images [12]. Additionally, the generalized autocalibrating partially parallel acquisition (GRAPPA) can also be implemented to shorten the running time [12].

Thus, the aims of this study were to investigate the ADC values of cervical IVDs using the latest RS-EPI technique (RESOLVE) and then to evaluate the relation between ADC values and gender, age, anatomical level, and Pfirrmann grade.

## 2. Materials and Methods

### 2.1. Ethics Statement and Study Sample

The study was approved by the institutional review board and informed consent about the purpose of this study and usage of the data was obtained from each participant before enrollment. The inclusion of patients was previously reported [13, 14]: patients with neck pain and upper numbness, including paresthesia. The patients who met following criteria were excluded: diabetes mellitus, serious systemic disease, trauma history of the spine, infection history of the spine, and back surgery.

Fifty-two patients were included in this study (26 males, 26 females; average age: 44.15 ± 14.77 years; range: 20–76 years; 260 discs in total) and 40 asymptomatic volunteers (22 males, 18 females; average age: 35.64 ± 10.65 years; range: 22–70 years; 200 discs in total). They were consecutively recruited from October 2015 to August 2016 and accepted traditional T2-WI, T1-WI, and DWI examinations of their cervical spines. All MRI tests were performed in afternoon to minimize diurnal changes of intervertebral disc [15].

### 2.2. Magnetic Resonance Imaging

Cervical spine (C2-C3 to C6-C7) imaging was performed on a 3.0 Tesla MRI scanner (Magnetom Skyra, Siemens Healthcare, Erlangen, Germany) with a 12-channel head-neck coil. The MR images were acquired using the following sequences: (1) sagittal and axial, turbo spin-echo (TSE) T2-weighted imaging (T2-WI): repetition time/echo time (TR/TE) = 3,000/96 ms; slice thickness = 4 mm; gap = 0.4 mm; field of view (FOV) = 260 mm × 260 mm; matrix = 320 × 240; number of slices = 8, band width = 284 Hz/Pixel. number of signal-intensity acquisition = 2. (2) sagittal TSE T1-WI: TR/TE = 3,000/96 ms, field of view = 260 mm × 260 mm, matrix = 320 × 240, slice thickness = 4 mm, intersection gap = 0.4 mm, number of slices = 8, band width = 284 Hz/Pixel, number of signal-intensity acquisitions = 2. (3) The sagittal diffusion-weighted images were obtained by using readout-segmented echo sequence. GRAPPA was also used in this RS-EPI DWI sequences with a factor of 2. The acquisition times were 2 min and 55 s for the RS-EPI DWI imaging. The imaging parameters of RS-EPI DWI were as follows: TR/TE = 4900/75 ms; slice thickness = 3 mm; gap = 0.9 mm; FOV = 220 mm × 220 mm; matrix = 192 × 192; average = 1; readout segments = 7; echo spacing = 0.34 ms; number of slices = 12; bandwidth = 651 Hz/Pixel; *b* value = 0 and 500 s/mm^2^; direction = 3.

### 2.3. Inter- and Intra-Observer Agreement Analysis

All images were analyzed twice with a 4-week interval in a random sequence to avoid recall bias. Inter-observer agreement analysis was carried out by comparing the initial responses of the 2 independent observers. Intra-observer agreement was evaluated by comparing the same observer's responses between two times on each case.

### 2.4. Measurement of ADC Values

**Figure 1:**
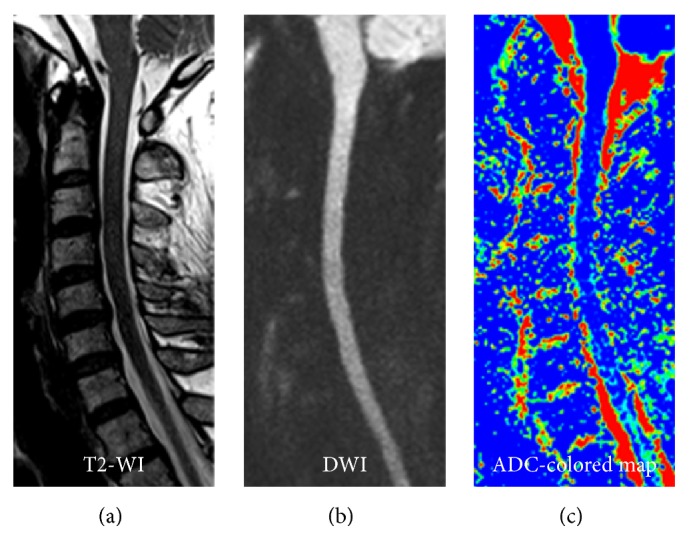
T2-WI, DWI, and ADC-colored map images of the cervical spine. (a) T2-WI image of the cervical spine, showing the anatomical information of the cervical spine and discs. (b) The diffusion-weighted image (DWI) of the cervical spine (*b* value = 0 and 500 s/mm^2^). (c) ADC-colored map of the cervical spine.

**Figure 2:**
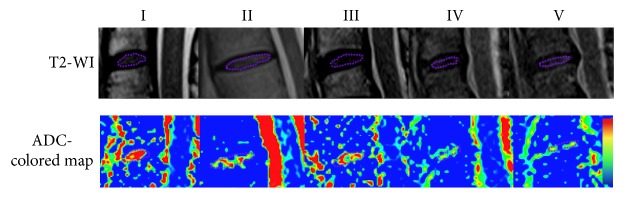
T2-WI and ADC-colored map of the cervical discs at each Pfirrmann grade and the ROI selection. According to the morphological shape of the disc on the T2-WI image, the ROI was manually delineated to cover the nucleus pulposus (NP) and then copied and pasted onto the ADC-colored map.

To measure ADC values, region of interests (ROIs) were manually drawn to define the nucleus pulposus (NP), according to different NP shapes of the cervical IVDs on T2-WI images. Then ROIs were copied and pasted onto the according ADC images (Figures 1 and 2). When inner NP was not distinguishable from outer annulus fibrosus in severely degenerative IVDs, the method reported by previous studies was adopted [13, 14, 16]. By using T2-WI as reference, regions of interest (ROIs) were manually drawn over the T2 map of the discs by an orthopedic consultant (GJ) with 20 years' experience reading MRI images. The geometrical center of the ellipses was defined to the intervertebral area ROIs only included the NP area. All ROIs were selected on the morphological images and transferred via “copy and paste” into the T2 maps ADC values were reported as mean ± SD.

### 2.5. Statistical Analysis

Statistical analysis was performed using SPSS 21.0 software (SPSS Inc., Chicago, IL). We tested intra-observer and inter-observer agreements by using *k* statistics for evaluating the reliability of Pfirrmann grading [14]. Student's *t*-test was used to compare ADC values between male and female at different anatomic levels. Pearson's rank correlation coefficient was used to evaluate the correlation between age and ADC values. Univariate ANOVA and post hoc tests were used to detect significance between ADC values of different degeneration grades. In addition, Welch's correction was used if heteroscedasticity was detected. Furthermore, Spearman's rank correlation coefficient was used to measure the correlation between ADC values and Pfirrmann grade. Finally, receiver operating characteristic (ROC) curve was plotted, and area under the curve (AUC) and the cut-off values were calculated. *P* < 0.05 was considered statistically significant.

## 3. Results

Pfirrmann grading results are given in Table 1, showing degenerative degree of all symptomatic participants' cervical discs. Based on Pfirrmann grade of IVDs, the intra-observer agreement was excellent to both observers (*k* = 0.793, *P* < 0.001), whereas the inter-observer test produced *k* values of 0.801 (*P* < 0.001). In the repeated evaluation 1 month after the first assessment, the agreement was also considered excellent using *k* statistics (*k* = 0.834, *P* < 0.001).

In general, the ADC values decreased from C2-C3 to C5-C6 (*P* < 0.05) and then increased from C5-C6 to C6-C7 (*P* < 0.05) (Table 2), such tendency could be found in both male and female patients. Additionally, the comparison between male and female patients yielded significance at each anatomical level from C2-C3 to C5-C6 (*P* < 0.05), showing cervical IVDs of male patients had significant higher ADC values than those of female patients (*P* < 0.05). At C6-C7, there was no statistical significance when comparing ADC values of males with those of females (*P* > 0.05), although the former showed higher values. In addition, negative correlation between age and the ADC value of NP was demonstrated either in the patient group (*r* = −0.790, *P* ≤ 0.001) or in the healthy group (*r* = −0.754, *P* ≤ 0.001).

**Figure 3:**
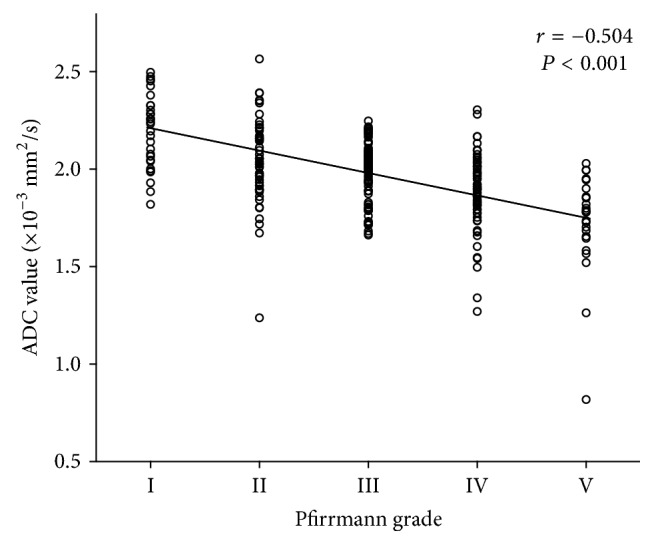
Negative correlation between the ADC value and the Pfirrmann grade. As shown in the graph, the ADC value decreased with higher Pfirrmann grade (*r* = −0.504, *P* < 0.001).

In both patient and volunteer group, the ADC values decreased with increasing degenerative grade from Pfirrmann grade I to V (*P* < 0.001) (Figure 2 and Table 3), and the ADC values were reversely correlated with Pfirrmann grades (*r* = −0.504, *P* < 0.001) (Figure 3). The overall ADC mean value of the volunteer group was significantly higher than its counterpart of the patient group (2.18 ± 0.18 versus 1.91 ± 0.23 × 10^−3^ mm^2^/sec, *P* < 0.05). In particular, at the each Pfirrmann grade, the patients showed lower ADC value than healthy volunteers (Table 3). In the volunteer group, five discs with Pfirrmann grade V were not considered in the statistical analysis since the small sample size.

As shown in Tables 4 and 5, the sensitivity, specificity, area under the ROC curve (AUC), and ADC cut-off value between different Pfirrmann grades of C2–C7 were calculated.

## 4. Discussion

In present observational study, we applied RESOLVE technique to investigate the ADC values of cervical discs in a symptomatic population. The differences between different genders, anatomical levels, and degenerative grades were analyzed; thereafter the correlation coefficients between the ADC value and age, anatomical level, and degenerative grade were investigated. Finally, the ROC curve was generated to give the cut-off ADC values of various Pfirrmann grades.

In this study, we found male patients had higher ADC values of the cervical IVDs at all five anatomical levels compared with female patients; four out of five comparisons yielded significance except at C6-C7. However, the relation between disc degeneration and gender was often debated. Evidence showed males are more vulnerable to musculoskeletal degenerative diseases, due to increased mechanical stress and occupational factors [17]. Nevertheless, elderly population displayed an opposite trend. By summarizing 98 studies, Wáng et al. [18] found that females have a higher prevalence of low back pain because of estrogen deficiency. It indicates that estrogen deficiency, as a systematic change of female, may also affect degeneration of cervical IVDs.

Also, we found that the mean ADC value decreased from 2.02 × 10^−3^ mm^2^/sec at C2-3 to 1.93 × 10^−3^ mm^2^/sec at C5-6, showing more caudal discs had lower ADC values than cephalic discs. This finding is consistent with the findings previously reported about lumbar discs [4, 19]. Moreover, in our research, we found that the IVDs at C6-C7 showed higher mean ADC value than the IVDs at C5-C6, at which the lowest mean ADC value was reached. The same finding was reported by Teraguchi et al. [20] in a large-scale epidemiology survey about cervical disc degeneration, in which they found the prevalence of disc degeneration was highest at C5-C6, followed by C6-C7 and C4-C5, due to its largest range of motion among all cervical discs [21].

In the present study we also investigated the influence of degenerative severity on the ADC value and found that mean ADC values of visually degenerative discs (Pfirrmann grades II–V) were lower than the data of lumbar discs reported by Kealey et al. [4] and Niinimäki et al. [22]. Such discrepancy may result from the differences of biochemical components and biomechanical burdens between cervical and lumbar discs and the different settings of the DWI sequences for data acquisition.

In this study, negative correlation was demonstrated between the ADC value and either age or degenerative grade in cervical discs. Similarly, Yu et al. [23] found in lumbar discs a significant negative correlation between ADC value and Pfirrmann grade. IVDs are nonvascular and nonsynovial structures with limited regenerative capacities. With degeneration, the content of proteoglycans and type II collagen in the NP will be lost and replaced with type I collagen [24]; this biochemical alternation will cause water content loss due to reduced hydrophilic matrix. Since the ADC value from the DWI MRI is highly sensitive to the change of the water molecule diffusibility, aforementioned biochemical change will eventually reduce water diffusion in the NP tissue and then result in lower ADC values.

Furthermore, it is important to note that a significance exists in the comparison of the ADC values between discs at Pfirrmann grades I and II. However, it was reported by Kealey et al. [4] that, in lumbar spine, no significance could be detected when they compared the ADC values of Pfirrmann grade I discs with those of Pfirrmann grade II discs. We reckon this contradiction may derive from dissimilar composition of the patient populations, since the mean age of subjects in our study was younger than that in the aforementioned study.

It is difficult to distinguish a painful degenerative disc from one disc with age-related physiologic changes [23]. Therefore, we recruited a well-defined asymptomatic population in the current study to inspect the effect of aging on the ADC value. However, we did not consider the equivalent age distribution of asymptomatic and symptomatic groups, which could introduce influence when we analyzed the differences between these two groups. Additionally, our study had a wider range of age than previous study [19]. Nevertheless, we also confirmed a negative correlation between age and the ADC value in symptomatic patients. Although the bias due to diverse age distribution could be uncertainty when we compared our findings to others' findings, but many researchers still declaimed the usefulness of DWI technique and proved the reliability of the ADC value at evaluating disc degeneration [4, 19, 25].

To figure out cut-off values of different Pfirrmann grades, ROC curves were plotted and the values of area under the curve (AUC) were calculated. In our study, the AUC values indicated moderate accuracy of ADC values at evaluation of degenerative degree of IVDs. Unlike traditional Pfirrmann grading system based on visual assessment, DWI technique is a quantitative method. Furthermore, we found that the ADC values were higher in the healthy population than the symptomatic patients at the same Pfirrmann grade, confirming the sensitivity of the ADC value at distinguishing the disc degeneration. This allows DWI MRI to be used for the assessment of disc degeneration in the clinical setting.

The advantage of this research lays in many aspects. Firstly, we used the RESOLVE technique for all DWI MRI tests, which currently is an advanced DWI technique of RS-EPI. As proved by several studies that the ADC value is correlated with biochemical content in the discs [4, 7, 22]; thus the ADC value can provide superior information about molecular and physiological alterations at early stage of IVDD before visual changes present. Therefore, the potential usages of this technique could be early identification of disc degeneration; continuous quantitative monitoring of disc degeneration or therapeutic effect of biological treatments dealing with disc degeneration.

Traditional SS-EPI sequence will produce significant magnetic susceptibility variation because of the spin dephasing [26]. Spin dephasing occurs during the relatively long echo trains for data collection from the whole *k*-space [26]. Using RS-EPI can avoid this problem with shorter length of the EPI readout for individual readout segment and thus shorter echo spacing and less distortion. With the integrated 2D navigator-echo acquisition introduced by Porter and Heidemann [12], the shot-to-shot motion-induced phase changes can be corrected during multishot acquisitions. In addition, this navigator can also control the real-time reacquisition of uncorrectable data by nonlinear phase correction [27], which is robust to correct the variations caused by minuscule motions between readout segments as well as rigid and nonrigid body motions [28].

However, there were some limitations in this study. Firstly, this was an observational study with a relatively small sample size. Thus, larger sample size of subjects and asymptomatic volunteers are needed to determine the clinical feasibility of this new MRI technique as a routine MRI test for patients with cervical disc degeneration. Secondly, many other MRI-based techniques, such as T1rho MRI, chemical exchange saturation transfer (CEST), T2 mapping, and T2 star mapping, also can reflect subtle changes of disc degeneration from different aspects [13, 14, 16, 29]. Although the effectiveness and sensitivity of these techniques were also confirmed by many studies, we did not compare these techniques with RS-EPI DWI in this study. Also, the comparisons between DWI and other MRI-based techniques should be investigated in the future for comprehensive understanding of DWI. Thirdly, subjectivity and bias still existed in our results because of manual delineation of the ROIs. Particularly, the boundary between the NP and the annulus fibrosus was not clearly distinguishable at IVDs with Pfirrmann grades IV and V. Fourthly, the present findings were not verified by histological and biochemical analysis.

## 5. Conclusions

Our study provided detailed data about ADC values and further demonstrated the negative correlation between the ADC value and either age or degenerative grade. All these findings suggest the ADC value from readout-segmented echo-planar DWI was a reliable and sensitive parameter to evaluate cervical disc degeneration.

## Figures and Tables

**Table 1:** Numbers of the discs at each Pfirrmann grade classified by gender and anatomical level in symptomatic volunteers.

Pfirrmann grade	Sex		Disc level					Total
	Male	Female	C2-C3	C3-C4	C4-C5	C5-C6	C6-C7	
I	15	14	10	6	5	2	6	29
II	35	20	17	11	10	6	11	55
III	57	31	15	20	22	19	12	88
IV	19	45	9	11	8	18	18	64
V	4	20	1	4	7	7	5	24
Total	130	130	52	52	52	52	52	260

**Table 2:** Mean ADC values of cervical spine sorted by sex and anatomical level (×10^−3^ mm^2^/sec).

Anatomical level	Sex		*P* value^*∗*^	Total
	Male	Female		
C2-C3	2.10 ± 0.17	1.94 ± 0.22	0.008	2.02 ± 0.21
C3-C4	2.05 ± 0.20	1.93 ± 0.25	0.034	1.99 ± 0.23
C4-C5	2.02 ± 0.18	1.86 ± 0.20	0.006	1.94 ± 0.21
C5-C6	2.01 ± 0.15	1.83 ± 0.26	0.003	1.92 ± 0.23
C6-C7	2.03 ± 0.17	1.94 ± 0.30	0.196	1.98 ± 0.25
Total	2.04 ± 0.17	1.90 ± 0.25		1.97 ± 0.23

^*∗*^ *P* values were calculated by comparing ADC values of IVDs between male and female patients.

**Table 3:** ADC values of asymptomatic and symptomatic volunteers according to Pfirrmann grade.

Pfirrmann grade	Symptomatic patients		Asymptomatic volunteers	
	Number	ADC mean value (×10^−3^ mm^2^/sec)	Number	ADC mean value (×10^−3^ mm^2^/sec)
I	29	2.19 ± 0.18	44	2.45 ± 0.11
II	55	2.05 ± 0.19^@^	52	2.32 ± 0.17^$^
III	88	1.98 ± 0.15^@#^	63	2.16 ± 0.20^$%^
IV	64	1.88 ± 0.19^@#∧^	36	1.94 ± 0.16^$%~^
V	24	1.72 ± 0.26^@#∧&^	5	1.86 ± 0.24
Total	260	1.91 ± 0.23	200	2.18 ± 0.18

NP: nucleus pulposus, ^@^compared with ADC values of grade I discs of symptomatic volunteers, *P* < 0.05; ^#^compared with ADC values of grade II discs of symptomatic volunteers, *P* < 0.05; ^∧^compared with ADC values of grade III discs of symptomatic volunteers, *P* < 0.05; ^&^compared with ADC values of grade V discs of symptomatic volunteers, *P* < 0.05; ^$^compared with ADC values of grade I discs of asymptomatic volunteers, *P* < 0.05; ^%^compared with ADC values of grade II discs of asymptomatic volunteers, *P* < 0.05; ^~^compared with ADC values of grade III discs of asymptomatic volunteers, *P* < 0.05.

**Table 4:** The results of ROC curve and cut-off values between adjacent Pfirrmann grades.

Pfirrmann grade	Sensitivity (%)	Specificity (%)	AUC	Cut-off value (×10^−3^ mm^2^/sec)
I versus II	51.70	83.60	0.693	2.22
II versus III	38.20	86.40	0.600	2.14
III versus IV	60.20	70.30	0.648	1.98
IV versus V	75.00	62.50	0.714	1.80

**Table 5:** ADC cut-off value of each Pfirrmann grade.

Pfirrmann grade	I	II	III	IV	V
ADC value (×10^−3^ mm^2^/sec)	>2.22	2.14–2.22	1.98–2.14	1.80–1.98	<1.80

## Supplementary Materials

Supplementary Materials Supplementary Table  1: classification of disc degeneration.
