# Supplementary material for: Apparent Diffusion Coefficient of Diffusion-Weighted Imaging in Evaluation of Cervical Intervertebral Disc Degeneration: An Observational Study with 3.0 T Magnetic Resonance Imaging
Source: Biomed Res Int. 2018 Feb 18;2018:6843053. doi: 10.1155/2018/6843053 (PMC5835286; doi:10.1155/2018/6843053)
Supplement: Supplementary Materials — Supplementary Table 1: classification of disc degeneration. [file 6843053.f1.pdf]

Supplementary Table 1. Classification of disc degeneration

Grade	structure	Distinction of nucleus and annulus	Disc height
I	Homogeneous, bright white	Clear	Normal
II	Inhomogeneous	Clear	Normal
III	Inhomogeneous, gray	Unclear	Normal to slightly decreased
IV	Inhomogeneous, gray or black	Lost	Normal to moderately decreased
V	Inhomogeneous, black	Lost	Collapsed disc space
